# Trend and spatial distribution of infectious diseases in pregnant women in the state of Paraná-Brazil*

**DOI:** 10.1590/1518-8345.2838.3160

**Published:** 2019-08-19

**Authors:** Larissa Pereira Falavina, Maicon Henrique Lentsck, Thais Aidar de Freitas Mathias

**Affiliations:** 1 Universidade Estadual de Londrina, Centro de Ciências da Saúde, Londrina, PR, Brasil.; 2 Universidade Estadual de Maringá, Departamento de Enfermagem, Maringá, PR, Brasil.; 3 Universidade Estadual do Centro-Oeste, Departamento de Enfermagem, Guarapuava, PR, Brasil.

**Keywords:** Communicable Diseases, Disease Notification, Pregnancy, Obstetric Nursing, Health Information Systems, Public Health, Doenças Infecciosas, Notificação de Doenças, Gravidez, Enfermagem Obstétrica, Sistemas de Informação em Saúde, Saúde Pública, Enfermedades Infecciosas, Notificación de Enfermedades, El Embarazo, Enfermería Obstétrica, Sistemas de Información en Salud, Salud Pública

## Abstract

**Objective:**

to analyze the trend and spatial distribution of some diseases that require compulsory notification in pregnant women.

**Method:**

ecological study, with data from the *National Notifiable Diseases* Surveillance *System,* of the incidence of the six most frequent diseases that, require compulsory notification, in pregnant women. The Prais-Winsten model was used to analyze the trend classified as stable, decreasing and increasing, according to macro-regions. For the spatial analysis, the incidences distributed in percentiles, in choropleth maps, by Health Regions were calculated.

**Results:**

the most frequent infections were syphilis, dengue, Human Immunodeficiency Virus, influenza, hepatitis and toxoplasmosis. Incidence increased by 30.8%, 30.4%, 15.4% and 2.6%, on average, for syphilis, toxoplasmosis, dengue and Human Immunodeficiency Virus, respectively. On average, the incidence of syphilis increased by 40.5% in Macro-regional North and 38% in Macro-regional Northwest. The spatial analysis showed, in the last four years, high incidence of dengue, syphilis and infection by Human Immunodeficiency Virus, which reached 180.2, 141.7 and 100.8 cases per 10,000 live births, respectively.

**Conclusion:**

there were increased incidences of infection in pregnant women due to syphilis, toxoplasmosis and Human Immunodeficiency Virus, with differences in their spatial distribution, indicating that these diseases should be a priority in the care of pregnant women in more affected regions.

## Abstract (pt)

**Objetivo:**

analisar a tendência e a distribuição espacial de algumas doenças de notificação compulsória em gestantes.

**Método:**

estudo ecológico, com dados do Sistema Nacional de Agravos de Notificação, das incidências das seis doenças de notificação mais frequentes em gestantes. O modelo de Prais-Winsten foi utilizado para a análise da tendência classificada em estável, decrescente e crescente, segundo macrorregiões. Para a análise espacial, foram calculadas as incidências distribuídas em percentis, em mapas coropléticos, por Regiões de Saúde.

**Resultados:**

as infecções mais frequentes foram sífilis, dengue, *Human Immunodeficiency Virus*, influenza, hepatites e toxoplasmose. A incidência para sífilis, toxoplasmose, dengue e *Human Immunodeficiency Virus* aumentou 30,8%, 30,4%, 15,4% e 2,6% ao ano, em média, respectivamente. Em média, ao ano, a incidência de sífilis aumentou 40,5% na Macrorregional Norte e 38% na Macrorregional Noroeste. A análise espacial mostrou, no último quadriênio, incidência elevada para dengue, sífilis e infecção pelo *Human Immunodeficiency Virus* que chegaram, respectivamente, a 180,2, 141,7 e 100,8 casos por 10.000 nascidos vivos.

**Conclusão:**

houve aumento da incidência de infecção em gestantes por sífilis, toxoplasmose e *Human Immunodeficiency Virus*, com diferenças em sua distribuição espacial, indicando que esses agravos devem ser prioridade no atendimento à gestante em regiões mais acometidas.

## Abstract (es)

**Objetivo:**

analizar la tendencia y la distribución espacial de algunas enfermedades de notificación obligatoria en gestantes.

**Método:**

estudio ecológico, con datos del Sistema Nacional de Agravios de Notificación, de las incidencias de las seis enfermedades de notificación más frecuentes en gestantes. El modelo de Prais-Winsten fue utilizado para el análisis de la tendencia clasificada en estable, decreciente y creciente, según macrorregiones. Para el análisis espacial, se calcularon las incidencias, distribuidas en percentiles, en mapas coropléticos, por Regiones de Salud.

**Resultados:**

las infecciones más frecuentes fueron sífilis, dengue, *Human Immunodeficiency Virus*, influenza, hepatitis y toxoplasmosis. Hubo aumento de la incidencia para sífilis, toxoplasmosis, dengue y Human Immunodeficiency Virus de 30,8%, 30,4%, 15,4% y 2,6% al año, en promedio, respectivamente. En promedio, al año, la incidencia de sífilis aumentaron un 40,5% en la Macrorregional Norte y un 38% en el Noroeste. El análisis espacial mostró, en el último cuadrienio, incidencias elevadas para dengue, sífilis e infección por el *Human Immunodeficiency Virus* que llegaron, respectivamente, a 180,2, 141,7 y 100,8 casos por 10.000 nacidos vivos.

**Conclusión:**

hubo aumento de la incidencia de infección en gestantes por sífilis, toxoplasmosis y *Human Immunodeficiency Virus,* con diferencias en su distribución espacial, indicando que esos agravios deben ser prioridad en la atención a la gestante en regiones más acometidas.

## Introduction

Decree 204, dated February 17, 2016, of the Ministry of Health (MH), establishes the diseases and aggravations of compulsory notification in Brazil and, among them, infectious diseases^1^, which are still part of the epidemiological profile of the population and, when they affect women during pregnancy, may compromise their health and that of the newborn.

Compulsory notification infectious diseases range from ancient diseases such as syphilis, dengue, Human Immunodeficiency Virus (HIV), Acquired Immunodeficiency Syndrome (Aids) and hepatitis, to recent infections, such as the Zika virus. The occurrence of infectious diseases may vary by region. Developing countries aggregate the majority of cases^2-4^ and in some regions of the world such as India, Africa and the Middle East, these diseases are still considered the leading cause of maternal death^3^.

A study with a population of pregnant women in a rural area of Ghana found a high prevalence of hepatitis B (16.7%) and malaria (10.6%)^2^. In the United States, the incidence of congenital syphilis increased from 8.4 per 100,000 in 2012 to 11.6 per 100,000 live births in 2014, reflecting the increase in disease among pregnant women^5^. In a municipality in Gabon, a country in the African continent, a study with 973 pregnant women found a prevalence of 2.5% for syphilis, 4.0% for HIV infection and 57.3% for toxoplasmosis^6^.

In Brazil, a cross-sectional study, which analyzed the rapid test records in pregnant women performed during prenatal care in the city of Maceió, identified a prevalence of 2.8% syphilis, 0.3% HIV infection and 0.4% Hepatitis B^7^. In the city of Niterói-RJ, a study carried out, also with serological tests records of pregnant women attended at a university hospital, found a prevalence of 1.5% for syphilis, 0.9% and 1.6% for hepatitis B and C respectively, and 5.8% for HIV infection^8^.

These studies show the profile of infectious diseases in gestation, especially those that are part of prenatal screening protocols, such as syphilis, HIV infection, toxoplasmosis and hepatitis B, recorded in the patient’s medical records or documents^7-9^. However, it can be seen, that the studies are not enough to present the joint analysis of the infectious diseases of compulsory notification occurred in pregnant women, mainly with the analysis of trend in recent period. It is also necessary to know the spatial distribution of infectious diseases to trigger preventive actions in identified geographical areas.

Studies of the behavior of these diseases over time and in the geographical space can contribute to the evaluation of public policies and control of infectious diseases in pregnant women. Thus, the objective of this study was to analyze the trend and spatial distribution of some infectious diseases of compulsory notification in pregnant women in the state of Paraná.

## Method

Ecological study^10^of the main diseases that require compulsory notification, in pregnant women, living in the state of Paraná, from January 1, 2007 to December 31, 2016.

**Figure 1:**
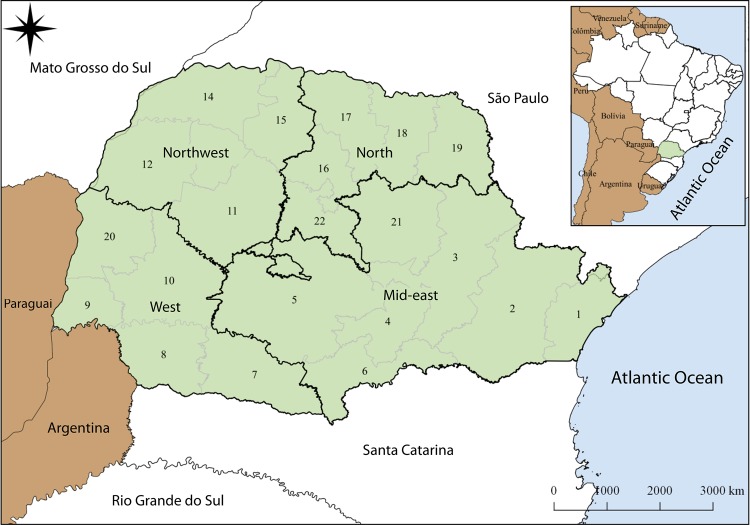
Regions* and Macro-Regions of Health in the state of Paraná *1 - Paranaguá, 2 - Curitiba, 3 - Ponta Grossa, 4 - Irati, 5 - Guarapuava, 6 - União da Vitória, 7 - Pato Branco, 8 - Francisco Beltrão, 9 - Foz do Iguaçu, 10 - Cascavel, 11 - Campo Mourão, 12 - Umuarama, 13 - Cianorte, 14 - Paranavaí, 15 - Maringá, 16 - Apucarana, 17 - Londrina, 18 - Cornélio Procópio, 19 - Jacarezinho, 20 - Toledo, 21 - Telêmaco Borba and 22 - Ivaiporã.

Paraná is one of the three states in the southern region of Brazil with an extensive border region with the states of Santa Catarina, São Paulo and Mato Grosso do Sul, with the countries of Argentina, Paraguay and the Atlantic Ocean. It has 399 municipalities and its estimated population in 2016 was 11,242,720 inhabitants^11^. The Human Development Index (HDI), released in the 2010 Census, was 0.749, the fifth highest among the states of the country^11^. The state of Paraná is divided into 22 Regions and four Macro-regions of Health (Central East, West, North and Northwest) responsible for health care management (Figure 1).

The study was carried out, with data from the National System of Notifiable Diseases (SINAN), which brings together all cases of compulsory notification diseases in Brazil. The database with all cases, suspected or confirmed, of diseases of compulsory notification in pregnant women was obtained by request at the electronic address of the Law on Access to Information (Protocol No. 25820002794201770 - 2017). This law (No. 12,527, of 2011) became effective, throughout the national territory in 2012 and regulates the right of access to public information, to any citizen.

The database with 42,040 records of diseases and injuries in pregnant women was received in June 2017. Of these, 153 residents were excluded in other states or with the field “State of residence” not completed and 10,525 relating to non-infectious diseases, totaling 31,362 notifications analyzed. In the database spreadsheet, columns were included for the code of 399 municipalities, for the Macro-regions and Health Regions of the pregnant woman place of residence.

The incidence of communicable diseases was analyzed in pregnant women (six main), per 10,000 live births, according to the year of notification, Regional and Macro-regional Health. For the calculation of the rates, the number of live births, from 2007 to 2015, was obtained from the Live Birth Information System (SINASC) through the electronic address of the Department of Informatics of the Single Health System (DATASUS). Data for the year 2016 was not available on DATASUS at the time of collection. Thus, they were donated by the 15th Health Regional of Paraná. The results were presented in figures and maps of the state of Paraná grouped in two triennia (2007-2009 and 2010-2012) and one quadrennial (2013-2016).

The trend analysis was performed for the entire state and Health Macro-regions using the Prais-Winsten model, which considers as dependent variable (Y) rates and independent variable (X) the year studied. This model is indicated for trend analysis, since it corrects the temporal autocorrelation of residues^12^, starting from the ecological assumption that the impacts can be influenced among themselves in the years of the time series. The smoothing of the rates for the time series was performed by the third order moving average. The analysis of the incidence scatter diagrams and the autocorrelation of the residuals allowed us to identify the behavior of the trend: stable (if p> 0.05); (p <0.05 and negative regression coefficient (β1)) and increasing (if p <0.05) and positive regression coefficient (β1)^12^. The regression coefficient of the Prais-Winsten model and the annual variation in the incidence of transmissible diseases in pregnant women in the period (in percentage) were estimated using the formula: (-1 + 10- ^ b) x100, since regression uses the logarithm of the rates (10 ^ b) (13). For trend analysis, Stata 13 software was used.

The spatial distribution of infection incidences of the six major notifying diseases in pregnant women according to the Health Regions was performed for the triennium 2007-2009 and the quadrennial 2013-2016 to compare possible differences between the beginning and the end of the period. For the choropleth maps, the color scale was used with lighter tones, indicating lower rates, and dark ones, higher rates. The rates were presented according to percentiles, that is, within the data matrix, each fraction corresponds to the respective percentile (zero, 25, 50 and 100).

The cartographic base of the state of Paraná, by municipalities, obtained from the electronic address of the Brazilian Institute of Geography and Statistics (IBGE), was used to create the cartographical maps and, from this, two cartographic bases were created for the Regions and Macro-regions of Health, using the “dissolve” tool, in the QGIS 2.8 software. It is noteworthy that the trend and spatial analyzes were performed for the six major notifying diseases in pregnant women in Paraná. This study was approved by the Research Involving Human Beings Ethics Committee of the Worker’s Hospital of the State of Paraná, with the opinion n. 2.156.066 / 2017, and obtained the exemption, of the Free and Informed Consent Term because it is search with secondary data.

## Results

Table 1 presents the results of the Prais-Wisten trend analysis for the six major infectious diseases in pregnant women. The rates for syphilis, toxoplasmosis, dengue and HIV/AIDS increased on average by 30.8%, 30.4%, 15.4% and 2.6%, respectively. In the North and Northwest Macro-regional, syphilis rates increased, on average, by 40.7% and 38%, respectively.

**Table 1:** Trend of the incidence of transmissible diseases in pregnant women * (per 10,000 live births), mean annual variation and confidence interval according to the Macro-regions of Health. Paraná, Brazil, 2007 to 2016

Macro-regions	2007-2009	Rate 2010-2012	2013-2016	Annual variation^†^	CI^‡^ (95%)	Trend
**Syphilis**						
Mid-east	16.6	41.5	118.0	31.5	26.6; 36.6	Crescent
West	24.6	37.1	116.2	24.9	14.5; 32.6	Crescent
North	7.9	23.8	105.6	40.7	32.3; 49.7	Crescent
Northwest	7.0	20.4	83.9	38.0	29.6; 46.6	Crescent
Paraná	15.2	34.6	110.3	30.8	24.7; 37.2	Crescent
**HIV** ^§^ **/Aids** ^‖^						Crescent
Mid-east	41.4	46.1	46.1	1.5	0.1; 2.9	Crescent
West	19.7	25.2	29.4	7.2	2.9; 11.7	Crescent
North	15.9	14.8	20.3	4.9	1.5; 8.5	Crescent
Northwest	17.3	18.4	25.5	5.0	1.1; 9.2	Crescent
Paraná	29.9	33.1	35.6	2.6	1.5; 3.6	Crescent
**Dengue**						
Mid-east	0.8	0.4	12.2	39.5	-18.4; 138.2	Stable
West	17.2	42.4	59.0	14.2	-1.1; 31.8	Stable
North	35.7	90.1	105.3	13.4	5.3; 22.2	Crescent
Northwest	44.0	40.0	132.8	9.5	-3.0; 23.6	Stable
Paraná	15.7	28.4	54.9	15.4	9.3; 21.9	Crescent
**Hepatitis**						
Mid-east	8.3	17.4	16.2	7.7	-0.6; 16.9	Stable
West	70.9	59.6	48.7	-4.9	-6.0; -3.8	Decrescent
North	14.4	18.1	15.3	-1.9	-8.1; 4.6	Stable
Northwest	37.4	24.3	12.7	-10.9	-19.0; -1.9	Decrescent
Paraná	24.5	26.0	21.1	-1.8	-4.7; 1.1	Stable
**Influenza**						
Mid-east	53.9	5.3	5.0	-5.4	-40.5; 52.9	Stable
West	90.5	8.2	4.3	-10.6	-47.7; 52.7	Stable
North	160.1	33.8	8.3	-4.0	-49.1; 81.1	Stable
Northwest	83.8	13.5	11.3	1.1	-38.1; 81.1	Stable
Paraná	82.1	11.7	6.4	-4.1	-43.1; 63.7	Stable
**Toxoplasmosis**						Stable
Mid-east	2.5	0.4	21.8	32.3	-22.8; 126.6	Stable
West	3.1	4.9	20.9	35.3	7.2; 70.9	Crescent
North	0.0	7.7	10.4	37.9	17.8; 61.3	Crescent
Northwest	7.5	15.5	27.8	19.8	13.9; 26.0	Crescent
Paraná	2.9	4.7	20.7	30.4	9.8; 54.9	Crescent

*Source: SINAN; †Average annual percentage change in the incidence calculated from the β_1_ of the Prais-Winsten generalized linear regression model; ‡CI - Confidence Interval; §HIV - Human Immunodeficiency Virus; ‖Aids - Acquired Immunodeficiency Syndrome.

**Figure 2:**
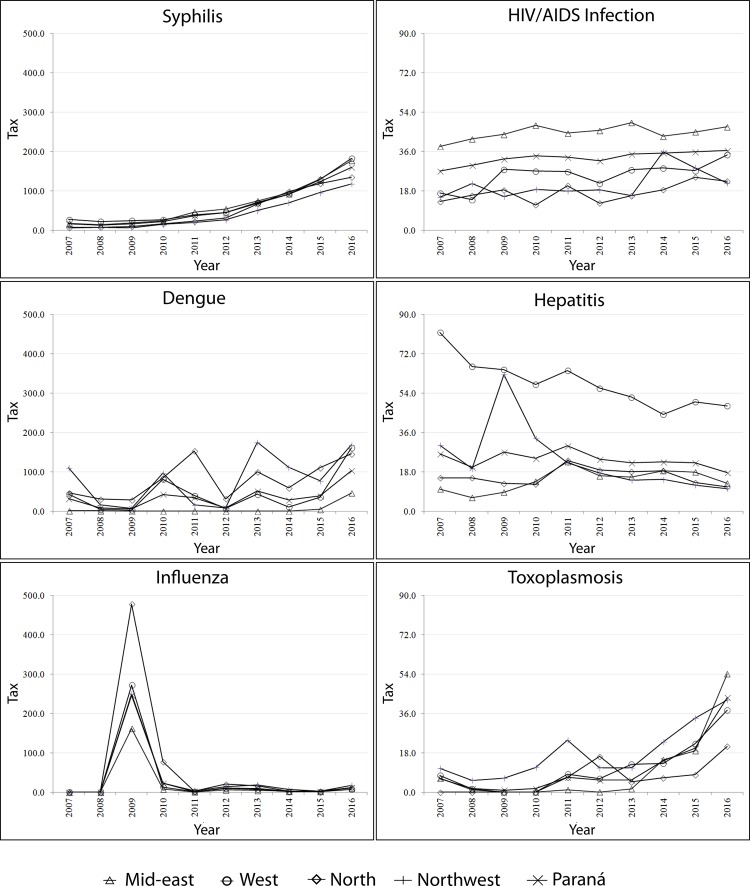
Incidence of communicable diseases in pregnant women (per 10,000 live births) according to Health Macro-regions. Paraná, Brazil, 2007 to 2016 *HIV - *Human Immunodeficiency Virus*; †Aids - *Acquired Immunodeficiency Syndrome*

When observing the trend lines for the four Health Macro-regions, the constant increase of the syphilis, from 2011 onwards, draws attention; of HIV infection, throughout the period; of toxoplasmosis, as of 2013 and the cyclical variation of dengue incidence (Figure 2).

**Figure 3:**
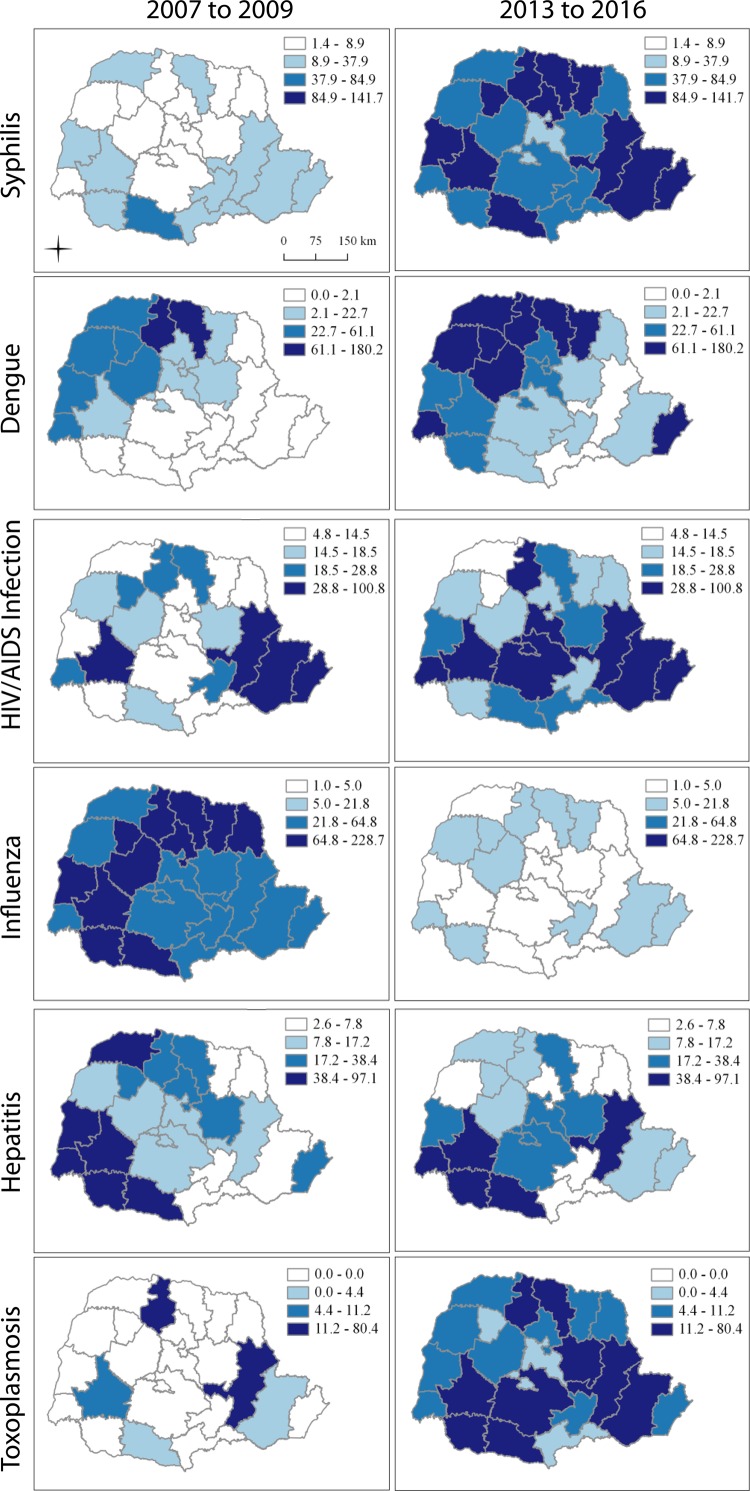
Spatial distribution of the incidence of transmissible diseases in pregnant women (per 10,000 live births) according to Health Regions‡. Paraná, Brazil, from 2007 to 2016 *HIV - *Human Immunodeficiency Virus*; †Aids - *Acquired Immunodeficiency Syndrome*; Health Regions described in Figure 1

Spatial analysis showed, in the last four years, higher incidence of dengue, syphilis and HIV / AIDS infection, reaching 180.2; 141.7 and 100.8 cases per 10,000 live births, respectively, with variations among Health Regions (Figure 3).

## Discussion

This study is innovative because it described the main infectious diseases in pregnant women reported in SINAN, identified and analyzed the trend and the spatial distribution of the six most frequent diseases in a period of ten years. The most frequent notifiable infectious diseases in pregnant women were: syphilis, dengue, HIV / AIDS, influenza, viral hepatitis and toxoplasmosis. Trend analysis showed increased rates of syphilis, HIV infection and toxoplasmosis, and the thematic maps illustrate this behavior by presenting rates in the first triennium and the last quadrennial.

Syphilis continues to be a public health problem for pregnant women worldwide, with serious consequences such as prematurity, fetal death and neonatal, which can be prevented with early diagnosis and treatment^14^. Recent studies on syphilis in pregnant women and newborns indicate that control measures are not sufficient and need to be intensified^14-16^. For quality prenatal care, special attention should be paid to all pregnant women, especially adolescents, those with 35 years of age or older, low schooling, race / black color, late onset or low number of visits of prenatal care^15,17^.

Improving health care for pregnant women is a priority in several regions of the world, however, a study conducted in a Health Regional Hospital in Brazil, through annual reports, identified that almost 70% of pregnant women in the public network did not have seven or more prenatal consultations, and only 49% and 50% had performed rapid syphilis and HIV tests, respectively^18^. These findings, complemented by those reported in this study, indicate urgency in the early capture of the pregnant woman, access and continuity of quality prenatal care. The nurse plays a fundamental role in the care of the pregnant woman with activities ranging from the Nursing consultation, requesting rapid tests and exams, guidelines and follow up in the diagnosis and early treatment of infectious diseases such as syphilis, HIV and toxoplasmosis^19^.

For the adequate control of infections, all professionals of the health teams must be able to provide care to pregnant women, but in a municipality in Paraná, the literature indicates that 22.5% of primary care professionals did not know the number of exams for syphilis during prenatal care; 28.4% did not know the conduct in their diagnosis; 41.1% did not know which control and follow-up procedures they had; 42% did not know the therapeutic scheme and 91.2% were unaware of the need for notification, a measure recommended since 2005^20^. It reinforces the role of the nurse, who is trained to guide the team on the necessary behaviors in relation to infectious diseases during pregnancy.

Although with lower rates, the constant and significant increase in the notifications of HIV infection in pregnant women was noticed, a result that agrees with that of other authors^21^. Analysis of AIDS cases in Brazil showed an increase in incidence, especially in the Southeast, South and Mid-West regions^21^. Factors associated with HIV infection include income, inadequate prenatal care, multiplicity of sexual partners, race/color black, homosexuality, unprotected sex, injecting drug use, blood transfusion, and accidents with sharp objects in health professionals^22-23^. In addition to these factors, women of reproductive age and, consequently, women in the gestational period, with a threat to the health of the newborn are also at risk for HIV infection, as identified in a study in Mozambique where children from infected mothers had a higher risk of prematurity, malnutrition, hospitalizations and death^24^.

In prenatal care, it is recommended to perform the rapid test at the first visit for all pregnant women: one in the third trimester and the other at the time of admission to labor^25^. This technology is one of the actions that help to reduce the vertical transmission of HIV infection, which has a target to be reached with an incidence of up to 50 cases per 100,000 live births^26^. Thus, it is necessary to intensify early prevention, diagnosis and treatment^27^, especially in regions with a greater number of cases identified in this study.

An important infection, which also presented a growing trend, was toxoplasmosis, which is part of the recommendations of the guide network of the Paranaense Mother Network program through the screening of all pregnant women in the first consultation or in the first trimester of pregnancy^25^. Because it is a disease that can cause intrauterine growth restriction, fetal death, prematurity, ocular and brain injuries, all pregnant women should be informed about prevention, which mainly involves care in handling food and contact with animal feces^28^.

In this study, other infections were reported, in pregnant women and, although they did not present an increasing tendency in the analyses, they deserve to be highlighted, such as influenza, which, when it occurs in pregnant women, can be serious and lead to hospitalization^29^. The pandemic, which occurred in 2009, showed that pregnant women are at increased risk for complications, and 5% of all deaths caused by infection worldwide occurred in women during gestation^30^. In Brazil, a study conducted in the state of Rio Grande do Sul during the pandemic identified 24 deaths of pregnant women due to influenza, in addition to eight fetal deaths^31^. In this study, it was clear that the majority of cases of influenza reported in pregnant women occurred in 2009, but in the following years there were still cases, ranging from 5.0 to 21.8 per 10,000 live births and also differences in their distribution space.

Severe cases of influenza still occur, as shown in a study in the state of California - United States, where 88% of pregnant women with severe influenza were hospitalized, 53% required mechanical ventilation, and 29% died^32^. As of 2009, the benefits of influenza vaccination were observed not only for pregnant women, but also for newborns during the first six months of life ^30^. Therefore, in Brazil, the vaccination schedule of pregnant women includes the influenza vaccine and it must be offered during prenatal care^25^.

In this study, the highest incidence of dengue in pregnant women occurred from 2013 to 2015, a result similar to that found by other authors for the southern region of Brazil^33^. A study on dengue in Brazil showed a nearly four-fold increased risk for pregnant women when compared to non-pregnant women^33^. It is important to highlight some preventive measures for dengue, such as encouraging the population on vector control, through campaigns and guidelines, focusing on regions with higher incidence.

This study innovates by adding relevant information that poses a risk to maternal and child health. The increase in the number of cases of some infectious diseases in pregnant women, reported in SINAN, is of concern, since it aggravates the health conditions of pregnant women. Likewise, the results showed where the cases of these infections are more concentrated, making it possible to identify the regions of the state of Paraná with worse indicators, which facilitates the implementation of preventive measures according to each reality.

These results are considered to have a direct and positive impact on Agenda 2030 for sustainable development, which, in its third objective, provides for the reduction of maternal mortality and preventable deaths in newborns, which are often caused by infections such as syphilis, HIV, and toxoplasmosis. In addition, this objective is aimed at combating the epidemics of infectious and transmissible diseases and, therefore, this study contributes to the reach of these actions in a way directed to the regions with greater risk of disease in pregnant women.

The interpretation of the results should consider that the study used secondary data that can be influenced both by the possibility of under reporting of cases of infectious diseases in pregnant women and by the quality and reliability of the information contained in SINAN. However, the quality of the database was not the object of study of this research and can occur in a differentiated way, between the regions of the state of Paraná.

There were areas and municipalities of the state with no or few reports of infectious diseases in pregnant women, which resulted in very low rates. To circumvent the effect of zero or very small rates, we chose to group the municipalities into larger geographic areas, such as the Regions and the Macro-regions of Health, and, for the trend analysis, the rates were smoothed through the mean mobile. It is worth mentioning that exploratory ecological studies, such as this one, are limited to population groups, so there can be no inference for individuals.

Finally, it is important to verify in future studies the factors that contribute to the incidence and increase of the incidence of compulsory notification diseases in pregnant women in the state of Paraná.

## Conclusion

The results of this study contribute to the knowledge about infectious diseases in pregnant women and indicate the existence of geographical inequalities related to women’s health, since the spatial distribution evidenced sites in the state of Paraná that should be prioritized, for the control of these infections.

The most prevalent infectious diseases in pregnant women were syphilis, dengue, HIV/AIDS, influenza, viral hepatitis and toxoplasmosis, with increased incidence of syphilis, HIV infection and toxoplasmosis evidenced by trend analysis. These results show that these diseases need to be a priority in the state of Paraná, imposing a challenge to managers, reviewing and restructuring public policies, and health professionals in the qualification of prenatal care.

## References

[1] Ministério da Saúde (BR) (2016). Portaria n. 204, de 17 de fevereiro de 2016. Define a Lista Nacional de
Notificação Compulsória de doenças, agravos e eventos de saúde pública nos
serviços de saúde públicos e privados em todo o território nacional, nos
termos do anexo, e dá outras providências.

[2] Volker F, Cooper P, Bader O, Uy A, Zimmermann O, Lugert R (2017). Prevalence of pregnancy-relevant infections in a rural setting of
Ghana. BMC Pregnancy Childb.

[3] World Health Organization (2017). HIV/AIDS.

[4] World Health Organization (2015). Sexually transmitted infections (STIs).

[5] Bowen V, Su J, Torrone E, Kidd S, Weinstock H (2015). Increase in incidence of congenital syphilis-United States
2012-2014. MMWR-Morbid Mortal W.

[6] Moukandja IP, Ngoungou EB, Lemamy GJ, Bisvigou U, Gessain A, Toure NFS (2017). Non-malarial infectious diseases of antenatal care in pregnant
women in Franceville, Gabon. BMC Pregancy Childb.

[7] Moura AA, Mello MJG, Correia JB (2015). Prevalence of syphilis, human immunodeficiency virus, hepatitis B
virus, and human T-lymphotropic virus infections and coinfections during pre
natal screening in an urban area Northeastern Brazilian
population. Int J Infect Dis.

[8] Vilte RMCV, Azevedo KML, Setúbal S, Oliveira SA (2016). Soroprevalence of toxoplasmosis, syphilis, hepatitis B, hepatitis
C, rubella, cytomegalovirosis and human immunodeficiency virus infection
among pregnant patients followed up from 2008 to 2012 at university hospital
Antônio Pedro, Niterói (RJ). J Bras Doenças Sex Transm.

[9] Ferezin RI, Bertolini DA, Demarchi IG (2013). Prevalence of positive sorology for HIV, hepatitis B,
toxoplasmosis and rubella in pregnant women from the northwestern region of
the state of Paraná. Rev Bras Ginecol Obstet.

[10] Almeida FN, Baretto ML (2011). Epidemiologia & saúde: fundamentos, métodos e aplicações.

[11] Instituto Paranaense de Desenvolvimento Econômico e Social (2016). Paraná em números.

[12] Antunes JL, Cardoso MRA (2015). Using time series analysis in epidemiological
studies. Epidemiol Serv Saúde.

[13] Santos MAS, Oliveira MM, Andrade SSCA, Nunes ML, Malta DC, Moura L (2015). Non-communicable chronic disease hospital morbidity trends in
Brazil, 2002-2012. Epidemiol Serv Saúde.

[14] Newman L, Kamb M, Hawkes S, Gomez G, Say L, Seuc A (2013). Global Estimatives of Syphilis in Pregnancy and Associated
Adverse Outcomes: Analysis of Multinational Antenatal Surveillance
Data. Plos Med.

[15] Domingues RMSM, Leal MC (2016). Incidence of congenital syphilis and factors associated with
vertical transmission: data from the Birth in Brazil study. Cad Saúde Pública.

[16] Manego RZ, Mombo-Ngoma G, Wittle M, Held J, Gmeiner M, Gebru T (2017). Demography, maternal health and the epidemiology of malaria and
other major infectious diseases in the rural department Tsamba-Magosti,
Ngounie Province, in central African Gabon. BMC Public Health.

[17] Nonato SM, MeloAPS, Guimarães MDC (2015). Syphilis in pregnancy and factors associated with congenital
syphilis in Belo Horizonte-MG, Brazil, 2010-2013. Epidemiol Serv Saúde.

[18] Baggio MA, Pereira FC, Guimarães ATB, Caldeira S, Vieira CS (2016). Rede mãe paranaense program: analysis of prenatal care in a
regional health district. Cogitare Enferm.

[19] Ministério da Saúde (BR), Protocolo clínico e diretrizes terapêuticas (PCDT) (2015). Atenção integral às pessoas com infecções sexualmente
transmissíveis.

[20] Lazanini FM, Barbosa DA (2017). Educational intervention in Primary Care for the prevention of
congenital syphilis. Rev. Latino-Am. Enfermagem.

[21] Sousa AIA, Junior VLP (2016). Spatial and temporal analysis of Aids cases in Brazil, 1996-2011:
increased risk areas over time. Epidemiol Serv Saúde.

[22] Aguiar BS, Buchalla CM, Chiaravalloti FN (2018). How many AIDS epidemics can occur in São Paulo
city?. Rev Saúde Pública.

[23] Mojola SA, Everett B (2012). STD and HIV risk factors among U.S. young adults: variations by
gender, race, ethnicity and sexual orientation. Perspect Sex Reprod Health.

[24] Rupérez M, González R, Maculuve S, Quintó L, López-Varela E, Augusto O (2017). Maternal HIV infection is an important health determinant in
non-HIV-infected infants. AIDS.

[25] Secretaria de Estado de Saúde do Paraná (2012). Linha Guia: Mãe Paranaense.

[26] World Health Organization, Joint United Nations Programme on HIV/AIDS (2016). Global Aids Up to date.

[27] Viana RB, Ferreira HC, Santos MLSC, Cabrita BAC (2013). Experiences of hiv-positive pregnant women in relation to nursing
care: a descriptive study. Ci Cuidado Saúde.

[28] Ministério da Saúde (BR), Secretaria de Atenção a Saúde, Departamento de Atenção Básica (2012). Atenção ao pré-natal de baixo risco.

[29] Eppes C (2016). Management of Infection for the
Obstetrician/Gynecologist. Obstet Gyn Clin N Am.

[30] Rasmussen SA, Jamieson DJ (2014). 2009 Influenza and Pregnancy – 5 Years Later. New Engl J Med.

[31] Silva AA, Ranieri TMS, Torres FD, Vianna FSL, Paniz GR, Sanseverino PB (2014). Impact on pregnancies in South Brazil from Influenza A (H1N1)
Pandemic: Cohort Study. PLOS ONE.

[32] Louie JK, Salibay CJ, Kang M, Glenn-Finer RE, Murray EL, Jamieson DJ (2015). Pregnancy and Severe Influenza Infection in the 2013-2014
Influenza Season. Obstet Gynecol.

[33] Nascimento LB, Siqueira CM, Coelho GE, Siqueira JB (2017). Dengue in pregnant women: characterization of cases in Brazil,
2007-2015. Epidemiol Serv Saude.

